# Implementation fidelity of village health and nutrition days in Hardoi District, Uttar Pradesh, India: a cross-sectional survey

**DOI:** 10.1186/s12913-019-4625-9

**Published:** 2019-10-26

**Authors:** Mira Johri, Louis Rodgers, Dinesh Chandra, Cybil Abou-Rizk, Eleanor Nash, Alok K. Mathur

**Affiliations:** 1 University of Montreal Hospital Research Centre (CRCHUM), Tour Saint-Antoine, Porte S03-910, 850, rue St-Denis, Montréal, (Québec) H2X 0A9 Canada; 2 Department of Health Management, Evaluation and Policy, School of Public Health, University of Montreal, Montréal, Québec Canada; 3 Department of Social and Preventive Medicine, School of Public Health, University of Montreal, Montréal, Québec Canada; 4 Independent Consultant, New Delhi, India; 5 Faculty of Arts and Sciences, University of Montreal, Montréal, Québec Canada; 6 Indian Institute of Health Management Research University, Jaipur, India

**Keywords:** Process assessment (health care), Health promotion, Maternal-Child health services, Child health services, Primary health care, Community health workers, Developing countries, India

## Abstract

**Background:**

Village Health and Nutrition Days (VHNDs) are a cornerstone of the Government of India’s strategy to provide first-contact primary health care to rural areas. Recent government programmes such as the Janani Suraksha Yojana (JSY) and Mission Indradhanush (MI) have catalysed important changes impacting VHNDs. To learn how VHNDs are currently being delivered, we assessed the fidelity of services provided as compared to government norms in a priority district of Uttar Pradesh.

**Methods:**

We fielded a cross-sectional study of VHNDs to provide a snapshot of health services functioning. Process evaluation data were collected via administrative sources, non-participant observation using a standardised form, and structured questionnaires. Questionnaires were designed using a framework to assess implementation fidelity. Key respondents were VHND participants, front-line workers involved in VHND delivery, and VHND non-participants (pregnant women due for antenatal care or children due for vaccination as per administrative records). Results were summarised as counts, frequencies, and proportions.

**Results:**

In the 30 villages randomly selected for inclusion, 36 VHNDs were scheduled but four (11.1%) were cancelled and one VHND was not surveyed. Vaccination and antenatal care were offered at 96.8% (30/31) and child weighing at 83.9% (26/31) of VHNDs. Other normed services were infrequently provided or completely absent. Health education and promotion were particularly weak; institutional delivery was the only topic discussed in a majority of VHNDs. The true proportion of any serious problem impeding vaccine delivery was 47.2% (17/36), comprising 4 VHND cancellations and 13 VHNDs experiencing vaccine shortages. Of the 13 incidents of vaccine shortage, 11 related to an unexpected global shortage of injectable polio vaccine (IPV). Over the 31 VHNDs, 37.8% (171 of the 452 scheduled beneficiaries) did not participate. Analysis of missed opportunities for vaccination highlighted inaccuracies in beneficiary identification and tracking and demand side-factors.

**Conclusions:**

The transformative potential of VHNDs to improve population health is only partially being met. A core subset of high-priority services for antenatal care, institutional delivery, and vaccination associated with high-priority government programmes (JSY, MI) is now being provided quite successfully. Other basic health promotion and prevention services are largely not provided, constituting a critical missed opportunity.

## Background

Village Health and Nutrition Days (VHNDs) are a key component of the Government of India (GoI)‘s strategy to provide first-contact primary health care to rural areas. Established by GoI in 2007, VHNDs are designed as a convenient service provision hub to make health services accessible to underserved rural communities. VHNDs are based on three important principles: (1) comprehensiveness and integrated service delivery: VHNDs bring together a large package of important health, nutrition, and sanitation services offered in a single location; (2) regularity and geographic proximity: the VHND is organized once per month in each village to facilitate regular service contact and ensure that rural residents do not have to incur costs related to time and travel to avail health services; (3) financial accessibility: all services offered at the VHND are provided free of charge [1].

VHNDs have an essential role to play in India’s strategy to attain the 2030 Sustainable Development Goals (SDGs), particularly Goal 2 (Zero Hunger), Goal 3 (Good Health and Well-Being), Goal 6 (Clean Water and Sanitation), and Goal 10 (Reduced inequalities) [2]. Services provided under the VHND umbrella are selected on the basis of their importance for population health based on India’s burden of disease and scientific evidence of impact. In addition to offering select basic health services, VHNDs are designed to help villagers learn about the protective and promotive aspects of health care and to foster appropriate health care seeking [1]. Core VHND services include those for reproductive, maternal, new-born and child health, tuberculosis and HIV treatment and control, and counselling for communicable disease prevention and health promotion [1].

VHND service delivery varies considerably among states and districts [3] and evolves constantly in response to changes in India’s health policy landscape. Two recent programs are especially salient: the “Janani Suraksha Yojana” (JSY), a conditional cash transfer program launched in 2005 to reduce maternal and neonatal mortality by increasing births in health facilities [4], and Mission Indradhanush (MI), launched in 2014 to fully immunise 90% of India’s infants against seven vaccine preventable diseases by 2020 [5]. Both programs include financial incentives for front line health workers in an attempt to boost service performance, and prioritize low-performing areas to improve equity in coverage and outcomes. Uttar Pradesh (UP) figured as a high-focus state for JSY and MI [5, 6].

In recent years, MI has catalysed a transformation of India’s immunization service delivery landscape and been a driving force for rapid changes impacting VHNDs. MI operated between April 2015 and July 2017 in 528 of India’s 640 (as per Census 2011 [7]) districts, including 44 districts in UP, but the estimated coverage increase of 6.7% was judged insufficient to achieve the programme objective of 90% full immunization coverage by 2020 [8]. From October 2017 to January 2018, an intensified MI strategy (IMI) was delivered in 173 lagging districts (including 59 districts in UP) and 17 urban areas with an accelerated timeline to fully immunise 90% of Indian infants against seven vaccine preventable diseases by 2018 [6, 8]. Vaccination coverage has increased with unprecedented rapidity under IMI, with an overall increase of 18.5% in full immunization coverage IMI districts [8]. Immunisation is one of the core services offered by VHNDs and MI and IMI policies to enhance health worker training, supervision, and monitoring, generate community demand for immunisation services, improve vaccine supply and immunisation data systems, and strengthen beneficiary tracking and micro-planning all impact VHND functioning, with potential spill overs, both positive and negative, to delivery of non-immunisation services [5, 6]. Although several important studies have contributed to our understanding of VHND functioning [3, 9–14], we are aware of no published study focusing on Uttar Pradesh, nor on VHND delivery in the context of MI and IMI.

Process evaluation aims to study the realities of program implementation to advance understanding of how and why public health interventions work [15]. “Implementation fidelity” refers to the degree to which an intervention is delivered as initially planned [16]. Fidelity assessment is an aspect of process evaluation that aims to understand and measure to what extent an intervention is being implemented as intended, and to shed light on what specific reasons have caused the success or failure of the intervention [16]. We fielded a study to assess the fidelity of services provided through VHNDs as compared to government norms in a high-priority district of Uttar Pradesh undergoing rapid health system transformation.

## Methods

### Design and setting

We conducted a cross-sectional study of VHNDs to provide a snapshot of health services functioning. Process evaluation data were collected from administrative sources, non-participant observations, and structured questionnaires using the following study procedures: The VHND schedule was accessed through administrative records. A team comprising 2 to 6 field staff was dispatched according to the scheduled location and time. A study form was used to record village characteristics, note whether the VHND had been held, and record reasons for VHND cancellation, if applicable. If the VHND took place, the field team undertook two days of data collection. On the first day, the entire VHND session was directly observed using a standardised record form. Individual structured interviews using questionnaires were conducted with front-line workers involved in VHND delivery and VHND participants. Administrative records of anticipated beneficiaries (due lists) were used to identify VHND non-participants. On the second day, survey teams traced all non-participants and administered questionnaires at their homes. Evaluators were independent of the intervention developers and implementors. Permission to conduct the study was obtained from Government of India officials prior to study initiation and all findings were shared after study completion.

The study was conducted from 2016 to 12-03 to 2017-03-04 in a rural district (Hardoi, population 4 million [17]) of the state of Uttar Pradesh (UP). In UP in 2015–16, female literacy was 61.0% (56.2% in rural areas) [18]; an estimated 46% of children 0–59 months of age were stunted, 40% were underweight, and 18% were wasted [18]; and 51.1% of children 12–23 months were fully immunized [18]. Hardoi is a poor performing area within Uttar Pradesh; Hardoi’s estimated under-5 mortality rate is 118 per 1000 (UP 90; India 57.3) [19, 20]. Hardoi district was a priority area for the JSY, MI and IMI initiatives. Our study was conducted towards the end of the MI initiative (the study was initiated after Phase 3 and completed 1 month before the start of Phase 4) and prior to introduction of the IMI strategy. Hardoi district is divided into 19 administrative blocks, of which a single block of approximately 200,000 inhabitants was selected for this survey. The block was selected in collaboration with district officials based on criteria related to logistics and relatively weak health indicators. The block name is not disclosed to protect respondent anonymity.

### Participants

The target populations for the assessment were: (1) VHND participants: Any adult attending to receive services for himself or herself, or for a child. (2) Front-line workers involved in VHND delivery (Assistant Nurse Midwives (ANM), Accredited Social Health Activists (ASHAs), and Anganwadi Workers (AWW)) and supervisors present at the VHND. (3) VHND non-participants: Defined as those listed on registers of pregnant women due for antenatal care services or children due for immunization, who did not attend the VHND session. Although many others are eligible for other VHND services (e.g. children due for weighing, adult men and women requiring family planning services, tuberculosis, or tobacco control), there is no beneficiary list for services other than antenatal care and immunization. It was therefore not possible to follow up this larger group of non-participants.

### Sample size and selection

We estimated the required sample size for a one group descriptive study by the method of calculating a confidence interval for a proportion. We wanted to ascertain the proportion of VHNDs in the sample experiencing problems that impeded vaccine delivery. We assumed that the true proportion of villages experiencing such problems was 10%, and that there was one VHND per village (a conservative assumption based on village population size). Based on these inputs and using a binomial (“exact”) calculation, we would require 30 villages to be able to detect a 95% confidence interval of 2.1 to 26.5% [21, 22].

Rural villages with less than 5000 inhabitants located in the selected block were eligible for inclusion. The sampling frame was informed by the 2011 Census of India [23]. We eliminated 7 villages with a population greater than 5000, 15 villages with 0 population, 4 urban villages, and 8 villages in which any field team member was well known on a personal basis. This left a sampling frame of 109 villages, from which 30 villages were selected at random using Microsoft Excel.

In each of the 30 villages, we aimed to interview at least 10 VHND participants, all front-line workers involved in VHND delivery, and all VHND non-participants. We conducted exit interviews with VHND participants at the VHND site. Front-line workers were interviewed at the VHND site. VHND non-participants were interviewed at their homes.

### Variables and data sources

The design of study instruments was informed by the implementation fidelity evaluation framework proposed by Carroll and colleagues [16] and extended by Hasson [24]. We adapted this framework to assess the fidelity of VHND delivery with reference to Indian government norms [1]. Study instruments considered four categories of factors: (1) adherence, defined as the time, place, and frequency of VHND delivery; (2) intervention reach, defined as “the proportion of the intended targeted audience that participates in an intervention” [15]; (3) intervention “dose” delivered, defined as “the number or amount of intended units of the intervention or intervention component delivered” [15]; and (4) intervention “dose” received, defined as “how the target population received the intervention” [15]. All data sources were quantitative. Time, place, and frequency of VHNDs were documented by direct (non-participant) observation using a structured record form. For intervention reach, we considered target audiences of pregnant women and children due for immunization, and used administrative records to compare the number of attendees recorded versus due to attend. Because administrative records in these settings may be incomplete, we also attempted to verify the true denominator of children due for vaccination by conducting a systematic search to identify households with children in the age range for receipt of primary vaccination (0–23 months) not recorded in the due list. We did not inquire about pregnant women who might have been missed, as inquiries about pregnancy status may be considered indiscreet. For dose delivered, we considered three sub-categories: presence of front-line workers and supervisors, availability of materials or equipment (comparing materials and equipment observed versus prescribed by the government), and services dispensed (documenting the variety, quality and adequacy of normed versus delivered preventive and curative health services, and health promotion and health education activities). For dose received, we considered the number of people who attended the VHND, their knowledge, satisfaction with services, and likelihood of returning, and reasons for non-participation among those who did not attend. We sought to identify potential moderating factors related to intervention complexity and context through policy documents [16, 24]. Information on further moderating factors such as participant responsiveness (perceived relevance and usefulness of VHNDs by front-line workers), recruitment (strategies used to approach and attract VHND participants), and strategies to facilitate high-quality delivery of the interventions (training, monitoring and quality control procedures for delivering VHNDs) was gathered through questionnaires administered to front-line workers [16]. The questionnaires used in this study were developed specifically for this study. Questionnaires were drafted in English and refined and finalised in Hindi. Study instruments were reviewed internally by an expert group, and field tested and revised prior to the VHND survey.

### Data management and statistical methods

Completed forms were stored in a secure locked location accessible only to designated staff. Data were entered by trained personnel into a data management software designed to protect against unauthorized access, use, modification, loss or theft [25]. To ensure confidentiality, data were anonymized.

We used descriptive statistics (counts, frequencies, proportions) to summarise data. Missing data were not imputed. Analyses were conducted in SPSS Statistics 24.

## Results

### Adherence and reach

**Fig. 1:**
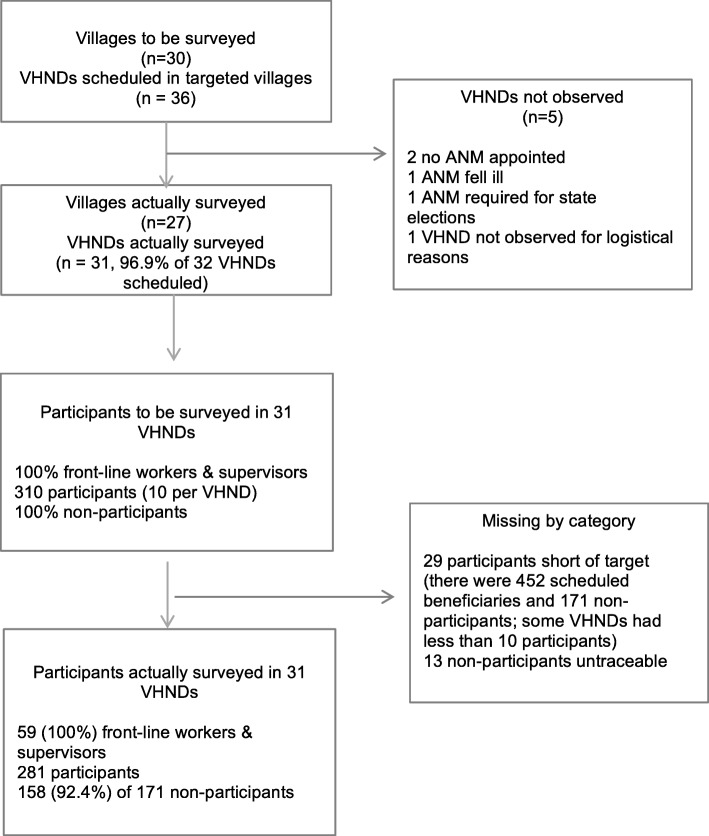
Flow diagram of process for study inclusion

In the 30 villages randomly selected for inclusion, 36 VHNDs were scheduled but four (11.1%) were cancelled. Our sample includes data from 31 VHNDs taking place in 27 villages. [Fig. 1].

The Village Health & Sanitation Committee was functioning in 66.7% (18/27) of villages surveyed, as reported by the ANM who is a committee member. Of the 31 VHNDs observed, 74.2% (23/31) were held in either an Anganwadi Center or a Sub-Center, and 96.8% (30/31) were held once a month. The due list of beneficiaries was not available in 12.9% (4/31) of the VHNDs, while in 6.5% (2/31) of the VHNDs the due list was judged to be incomplete as additional children not on the administrative due list were identified by the field staff. Vaccine shortages (vaccine unavailable, expired, or insufficient) were recorded in 41.9% (13/31) of VHNDs. Notably, 11 of the 13 VHNDs experiencing vaccine shortages encountered difficulties related to injectable polio vaccine (IPV). [Table 1] The true proportion of any serious problem impeding vaccine delivery was 47.2% (17/36), comprising 4 VHND cancellations and 13 vaccine shortages. Over the 31 VHNDs, 37.8% (171 of the 452 scheduled beneficiaries as per available due lists) did not participate. The number of non-participants ranged from 0 to 13 per village. Table 2 presents respondent characteristics.

**Table 1:** Problems in vaccine supply

Village ID	Not available	Shortage	Expired
1	IPV		
2	IPV		
3	IPV	PENTA	
7	IPV		
12	IPV		
15			IPV
18	IPV		
19	IPV		
20	OPV + IPV		
26	IPV		
29		PENTA	
30	IPV		

IPV – injectable polio vaccine

PENTA – pentavalent vaccine

OPV – oral polio vaccine

**Table 2:** Survey respondents^1^

	Personnel involved in VHND service delivery (*N* = 59) ^2^	VHND Participants (*N* = 281)	VHND Non-Participants* (*N* = 171)
Sex			
Female	59 (100.0)	234 (83.6)	81 (46.8)
Religion			
Hindu	–	266 (95.0)	162 (94.8)
Muslim	–	14 (5.0)	9 (5.3)
Category			
Assistant Nurse Midwife (ANM)	17		
Anganwadi Worker (AWW)	19		
ASHA	22		
ICDS Supervisor	1		

^1^All information given as n (%)

^2^Information on religion was not collected from service delivery personnel

^3^Information on the sex and religion of non-participants was taken from administrative records and verified in person where possible; 13/ 171 (7.6%) were untraceable

### Intervention “dose” delivered

#### Presence of front-line workers and supervisors

Of the 31 VHNDs observed, an average of 2.6 (range 1 to 5) front-line workers were present per VHND. The proportions of front-line workers present by category were: ANM 100% (31/31), ASHA 83.9% (26/31), Anganwadi Worker (AWWs) 74.2% (23/31). An Integrated Child Development Services (ICDS) supervisor was present at one VHND (3.2%; 1/31); no other type of supervisor was in attendance.

#### Availability of materials/ equipment

Among the 31 VHNDs observed, some items were always or mostly present, such as mother and child protection cards (100%; 31/31), stethoscopes (96.8%; 30/31), inch tapes (96.8%; 30/31), or weighing scales (90.3; 28/31). Other items were observed less frequently, such as zinc tablets (67.7%; 21/31), contraceptive pills (58.1%; 18/31), condoms (48.4%; 15/31), and ORS packets (45.2%; 14/31). Some required items were seldom found, such as soap (29%; 9/31), hand gloves (25.8%; 8/31), examination Tables (22.6%; 7/31), and referral cards (6.5%; 2/31).

#### Services and activities delivered

Vaccination and antenatal care were the services most frequently offered at the 31 VHNDs surveyed, followed by Vitamin A distribution and weighing. [Table 3] Other services were infrequently provided or completely absent. The most often discussed health education topic was institutional delivery, followed by registration for the JSY scheme. Many topics recommended in the Government of India VHND guidelines were not discussed with any participant in this sample.

**Table 3:** Overview of services provided

Activity or service^1^	# VHNDs (*N* = 31)	% VHNDs
Activity or Service		
Vaccination (children)	30	96.8
Antenatal care check-ups (pregnant women)	30	96.8
Vitamin A supplements (children)	27	87.1
Weighing (children)	26	83.9
Antenatal care registration	24	77.4
Tuberculosis treatment	5	16.1
Malnutrition management	4	12.9
Identification of anaemia	4	12.9
Distribution of oral rehydration salts	3	9.7
Identification of disabilities	0	0
Provision of condoms and oral contraceptive pills; external referrals	0	0
Identification of tuberculosis	0	0
Health promotion topics discussed		
Institutional delivery	26	83.9
Registration for the Janani Suraksha Yojana	19	61.3
Danger signs during pregnancy	14	45.2
Exclusive breastfeeding	14	45.2
Post-natal care	10	32.3
Nutrition	7	22.6
Care during diarrhoea and home management	4	12.9
Age at marriage	1	3.2
Dangers of sex selection	1	3.2
Weaning and complementary feeding	0	0
Care during acute respiratory infections	0	0
Prevention of malaria, tuberculosis, and other communicable diseases	0	0
Prevention of HIV/AIDS	0	0
Prevention of reproductive and sexually transmitted infections	0	0
Importance of safe drinking water	0	0
Personal hygiene	0	0
Household sanitation	0	0
Education of children	0	0
Disease outbreak	0	0
Disaster management	0	0

^1^ These are activities or services specified in the government of India VHND guidelines^6^

^2^ These are topics discussed with at least one participant at a given VHND

### Intervention “dose” received

#### VHND participants

VHND participants appeared satisfied with services received: 98.6% (277/281) planned to attend the next VHND and 94% (264/281) had a good or very good satisfaction level regarding the work of the ANM. Despite this, a majority of community respondents (56.6%; 159/281) declared that they did not know the role or purpose of VHNDs.

#### VHND non-participants

Of the 171 non-participant households, 13 dwellings were found locked and no information was gathered concerning reasons for non-participation. For the remaining 158 households, 68 households (43.0%) were absent for an extended period of time according to neighbours. The remaining 90 households directly provided reasons for not participating in the VHND. Table 4 summarises reasons for non-participation.

**Table 4:** Reasons for failure to participate in the VHND^1^

Reasons for non-participation	# Households (*N* = 158)	%
Entire household absent for extended period of time^2^	68	43.0
Household had not received information for the VHND	31	19.6
Family members all too busy to bring child	23	14.6
Child was unwell	16	10.1
Family problems	6	3.8
Distance (too far)	4	2.5
Fear of side effects	3	1.9
Felt no need for the VHND	2	1.3
Vaccine stock out	2	1.3
Vaccinated at a private clinic	2	1.3
Cultural of religious beliefs	1	0.6

^1^ 13 of the 171 non-participants could not be traced

^2^ Information provided by neighbours

### Additional factors

#### Frontline worker responses

Virtually all frontline workers reported that VHNDs were important (96.6%; 56/58), that they had received the proper training to deliver VHNDs (96.6%; 56/58), and that they had sufficient time and motivation to deliver VHNDs (96.6%; 56/58)). All (100%; 22/22) ASHA workers felt that financial incentives improved their performance. Of those who received training, 98.2% (55/56) stated that the training had been useful. The reported average duration of training was 9 ½ hours and 86.2% (50/58) wished to receive additional training. Vaccination (43.1%; 25/57), disease prevention (21.1%; 12/57), and general VHND preparation (14.0%; 8/57) were the most popular training topics mentioned.

#### Vaccine safety

Some elements potentially important for vaccination safety were not in place. None of the VHND beneficiaries ((0%; 0/31) were advised to wait in case of an adverse reaction following vaccination. None (0%; 0/31) of the VHNDs had an epinephrine kit in case of anaphylaxis.

## Discussion

VHNDs are critical initiatives to improve service delivery and population health in rural India. Designed to offer a comprehensive package of preventive, promotive and curative health services, VHNDs form a key element of the Government of India’s commitment to the advancement of all citizens, including those from marginalised and vulnerable communities [1]. Our study provides a comprehensive snapshot of health services functioning to assess the fidelity of services provided through VHNDs as compared to government norms. Our findings underscore a substantial discrepancy between the stated aims of the VHND framework and their implementation in practice. Briefly put, the tremendous potential of VHNDs is only partially met as only a subset of services is currently provided with high fidelity.

In recent years, the Government of India has made considerable efforts to strengthen antenatal care and institutional delivery services for pregnant women through the JSY program, [26] and immunization for pregnant women and children through MI [5] and IMI [6]. These services are now being provided quite successfully through VHNDs in our study area. VHNDs were generally delivered as planned, although 11% of sessions were cancelled due to unavailability of the ANM. Vaccination for children and antenatal care services for pregnant women were available in 97% of VHNDs. Although almost half of the VHNDs observed in our study were affected by vaccination shortages, virtually all (85%) of the incidents related to an unexpected global shortage of injectable polio vaccine [27, 28]. Vaccine supplies are generally adequate [29]. Child weighing promoted by the ICDS program is also performed in over 80% of VHNDs. However, a range of other key evidence-based services for population health that fall under the responsibility of VHNDs (such as promotion of improvements in water, sanitation, and hygiene practices, nutritional counselling, prevention, recognition and management of key illnesses such as diarrhoea, pneumonia, and mosquito-borne diseases, and tobacco control efforts) are largely not provided. Health education and promotion were particularly weak areas; the only topics discussed in a majority of VHNDs were institutional delivery and registration for the JSY program.

Several process evaluations of VHNDs in India have been conducted [3, 9–14], showing numerous points of convergence with our results: key messages surrounding vaccination were absent, presence of supervisors at VHNDs was low, and important services and materials were missing [9–11, 13, 14].. None of the other VHND evaluations take place in Uttar Pradesh, our study location. Moreover, only one other study documented the views of service providers and beneficiaries, [12] and none has focussed on the perspective of non-participants. Capturing these under-studied points of view is essential to understanding potential gaps in programme performance. Moreover, the timing of our study in relation to major policy initiatives such as JSY and MI is particularly salient.

Our findings show that, in Hardoi district at this juncture, antenatal care and vaccination for pregnant women and children have largely come to define the VHND platform. The policy context has played a fundamental role in shaping the emergence of this constellation of core VHND services. Launched in 2005, the JSY programme implemented conditional cash transfers to encourage births in health facilities. The JSY programme has had a significant effect on increasing antenatal care and in-facility births, and in improving neonatal survival [26]. Catalysed by the launch of Mission Indradhanush in early 2015, vaccination services in Hardoi district have improved decisively in recent years, with full immunization coverage in children aged 12–23 months rising from 26.5% in 2007–2008 [30], to 39.1% in 2015–2016 prior to our study, [31] to an estimated 65.9% (95% CI: 62.0–69.8%) in 2018 at the end of the IMI strategy [8, 32]. These unprecedented gains are the fruit of intensive structured investments in the building blocks of effective immunisation systems, such as strategic and operational planning, information systems, community support and demand generation, and vaccine supply and cold-chain management [8, 29, 33]. Extensive efforts have been made to ensure the quality of vaccination delivery by frontline workers, including training, supervision, rigorous external monitoring, and pay-for-performance incentives [5, 6, 8]. Gaps in the immunisation system remain, including three highlighted by our study.

1. *Vaccine safety*. At the time that this fidelity assessment was conducted, the GoI VHND guidelines did not require that an epinephrine kit be present at the VHND. Instead, each VHND is paired with a primary health centre that could provide emergency care. This is not an appropriate solution because, in this locale, distances are such that a child with anaphylactic shock would not reach the health centre in time to prevent death or severe disability. Second, communication concerning vaccination and especially vaccine safety were found to be absent or weak. The Government of India has recently revised guidelines for vaccine safety.
2. *Beneficiary identification and tracking*. We found that administrative due lists were highly inaccurate. In this resource-poor area, out-migration by families in search of employment is common and administrative due lists were substantially inflated, with almost half of those listed as non-participants (47%; 81/171) untraceable or non-resident. We also found two instances of families with children in the age range for primary vaccination who were not on the due list and inquired about the reasons. The families perceived that they were excluded due to differences with the ASHA workers. The ASHAs were reluctant to include families on the due list perceived as likely to be noncompliant. Systematic improvements to beneficiary tracking including door-to-door headcounts were prioritised as a part of IMI [6, 8].
3. *Missed opportunities for vaccination*. Among the non-participants interviewed, five reasons accounted for 91% of absenteeism: the entire household was absent for an extended period; the household had not received information about VHNDs; family members were too busy to bring the child; the child was unwell; and family problems. Future interventions should address these missed opportunities for vaccination by tailored approaches such as improving logistics, coordination, and information so that families temporarily away from their homes do not miss antenatal care services or a vaccine dose, striving to increase awareness among families about the need for timely vaccination so as to ensure that it is a health priority, and informing parents about vaccination being safe during a minor illness [34].

Our study has several strengths. This evaluation was supported by the state and district governments who ensured collaboration from those involved in service delivery. The survey was designed based on an established process evaluation framework and included information from VHND providers, participants, and non-participants. It used quantitative methods to measure key process variables to provide a comprehensive portrait of current VHND functioning [35]. Questionnaires were pre-tested and revised prior to being fielded by experienced survey teams.

Our study also had limitations. (1) We did not use qualitative research methods. We decided to focus on quantitative methods for several reasons: (i) The aims and methods of process evaluation must be tailored to the key evaluation questions held by intervention stakeholders [35]. For this study, the key stakeholders were government officials at district, state, and central levels responsible for the delivery of health services. Quantitative methods are generally used to shed insight into “what is delivered” and to test pre-hypothesised mechanisms of impact and contextual moderators, while qualitative methods are useful to capture unanticipated or adaptive changes in implementation, experiences of the intervention, and to yield insights into how and why interventions were delivered as they were [35]. VHNDs are a universal programme and the policy priority of the Government of India at a time of rapid transition was to gain insights into overall performance; (ii) In-depth interviews with frontline workers would have been desirable, but infeasible at this programme juncture. MI and IMI operated in mission mode to achieve time-bound objectives. ANMs and ASHAs were under extremely high pressure from supervisors to achieve performance targets and frequently assessed by external monitors conducting random checks. Under the circumstances, we did not believe that they would feel comfortable to provide honest answers. Moreover, transitions are by nature difficult and government staff at all levels were working with great dedication to achieve mission aims. We felt that it was inappropriate as external evaluators to question employees about working conditions at this juncture and that attempting to do so would jeopardise working relationships; (iii) at the beneficiary level, we anticipated that the number of non-participants would be large. As responses from VHND non-participants have not been previously studied, we wanted to present a comprehensive portrait using standard questions to assess reasons for missed vaccinations. In sum, we believe that the quantitative approach taken by our study was responsive to the key questions and concerns of evaluation stakeholders and that we were able to provide novel, credible and actionable findings related to implementation fidelity (what is actually delivered versus what is normed). Qualitative research offering an in-depth exploration of health worker and target beneficiary perspectives is an important area for future work. (2) We were unable to survey one VHND due to logistical constraints. (3) We observed the presence or absence of equipment but did not examine its quality. For example, we did not have a formal procedure to test weighing scales but observed that scales were often not working or highly inaccurate. (4) We attempted to verify the completeness of administrative due lists but our approach was limited as, in each village, our search was conducted on a single day and we did not perform a complete door-to-door census. Our results could underestimate the true magnitude of under-registration. (5) We attempted to trace all VHND non-participants but beneficiary lists exist only for antenatal care and vaccination; we have no information on reasons for non-participation for other services (which are largely not offered). Together, points 3, 4 and 5 suggest that our portrait of VHND functioning is likely to be optimistic. (6) Our results may be helpful in understanding the experiences of high-focus geographies for the JSY, MI and IMI programmes; whether results are generalizable beyond our study district is unknown.

## Conclusions

In this low-performing district of Uttar Pradesh, VHNDs have undergone a rapid transformation. Previously weak health services have been reinforced and a set of high-priority services related to antenatal care and immunization is now delivered with good fidelity. This is at once a tremendous achievement and a dilemma. These successes have been achieved by simplifying the VHND model to focus on a core subset of biomedical services whose delivery is readily measured and monitored, aligned with existing health worker skill levels, incentivised by pay-for-performance mechanisms, and closely tied to quantifiable health outcomes such as maternal, neonatal, and child survival.

The Sustainable Development Goals framework as translated by the Global Strategy for Women’s, Children’s and Adolescents’ Health (2016–2030) calls for a paradigm shift towards a holistic agenda encompassing three objectives: “Survive” (end preventable deaths), “Thrive” (ensure health and well-being) and “Transform” (expand enabling environments) [2, 36]. As designed, VHNDs are aligned with these goals. The VHND service package stipulated by the Government of India includes a wide range of evidence-based health interventions considered essential for health promotion, maintenance, and disease prevention, and not widely accessible from any other source [1]. However, as our study highlights, it has been difficult in practice to offer many of these services, which are often by their nature more challenging to deliver, less easily monitored, and operate through complex behavioural pathways only loosely tied to quantifiable health outcomes.

The dilemma facing policymakers is that the same approaches that have contributed to improved delivery of high-priority services cannot easily be extended to currently underprovided VHND services within the current health system organization. For example, responsibility for basic health education and promotion rests with the ASHA worker, but she receives little training for these tasks, no salary, and is paid only through incentives related to antenatal care, institutional delivery, and child vaccination activities. Performance-based incentives for health workers may result in neglect of non-incentivised tasks, [37] but only tasks that can be easily monitored can be linked to pay-for-performance incentives.

VHNDs were designed as an essential health package offering a guaranteed minimum to rural residents. While the original design may have been aspirational, the policy choices that have led to a narrowing of focus echo larger global debates. Since the 1978 WHO Alma Ata Declaration on Primary Health Care, the merits of comprehensive versus selective primary healthcare approaches have been hotly contested [38]. Proponents of the selective approach emphasise the importance of focussing evidence-based, low-cost solutions (such as antenatal care, institutional delivery, and immunisation) on high-priority problems (such as maternal and child mortality) to achieve interim gains [38]. Proponents of the comprehensive approach view health as a human right and highlight the responsibilities of governments to address the root causes of ill health, including social and structural determinants, to permit sustainable progress [38]. Inspired by the SDG vision to leave no one behind, the 2018 Astana Declaration on Primary Health Care renews a commitment to meeting all people’s health needs across the life course through comprehensive preventive, promotive, curative, rehabilitative services and palliative care [39]. In order for these goals to become reality, a strategic roadmap tailored to country health and development contexts that unites a holistic vision of health with a sound methodology and awareness of financial constraints, is required [38]. For India and other developing countries, the challenge of how to deliver the expanded and more complex range of services required to support holistic SDG health goals is a critical one.

## Data Availability

All data from this study are available from the corresponding author. The Hindi version of the questionnaire is available from the corresponding author upon reasonable request.

## Abbreviations

ANM: Assistant Nurse Midwives
ASHA: Accredited Social Health Activist
AWW: Anganwadi Workers
GoI: Government of India
ICDS: Integrated Child Development Services
IMI: Intensified Mission Indradhanush
JSY: Janani Suraksha Yojana
MI: Mission Indradhanush
SDG: Sustainable Development Goals
UP: Uttar Pradesh
VHND: Village Health and Nutrition Days
